# Ångström- and Nano-scale Pore-Based Nucleic Acid Sequencing of Current and Emergent Pathogens

**DOI:** 10.1557/adv.2020.402

**Published:** 2020-12-01

**Authors:** Britney A. Shepherd, Md Rubayat-E Tanjil, Yunjo Jeong, Bilgenur Baloğlu, Jingqiu Liao, Michael Cai Wang

**Affiliations:** 16 Department of Medical Engineering, University of South Florida, 4202 E. Fowler Avenue, Tampa, Florida 33620 USA; 26 Department of Mechanical Engineering, University of South Florida, 4202 E. Fowler Avenue, Tampa, Florida 33620 USA; 36 Centre for Biodiversity Genomics, University of Guelph, 50 Stone Road East, Guelph, Ontario N1G2W1 Canada; 46 Department of Systems Biology, Columbia University, 1130 St. Nicholas Avenue, New York, New York 10032 USA

## Abstract

State-of-the-art nanopore sequencing enables rapid and real-time identification of novel pathogens, which has wide application in various research areas and is an emerging diagnostic tool for infectious diseases including COVID-19. Nanopore translocation enables de novo sequencing with long reads (> 10 kb) of novel genomes, which has advantages over existing short-read sequencing technologies. Biological nanopore sequencing has already achieved success as a technology platform but it is sensitive to empirical factors such as pH and temperature. Alternatively, ångström- and nano-scale solid-state nanopores, especially those based on two-dimensional (2D) membranes, are promising next-generation technologies as they can surpass biological nanopores in the variety of membrane materials, ease of defining pore morphology, higher nucleotide detection sensitivity, and facilitation of novel and hybrid sequencing modalities. Since the discovery of graphene, atomically-thin 2D materials have shown immense potential for the fabrication of nanopores with well-defined geometry, rendering them viable candidates for nanopore sequencing membranes. Here, we review recent progress and future development trends of 2D materials and their ångström- and nano-scale pore-based nucleic acid (NA) sequencing including fabrication techniques and current and emerging sequencing modalities. In addition, we discuss the current challenges of translocation-based nanopore sequencing and provide an outlook on promising future research directions.

### INTRODUCTION

Emerging nanopore translocation technologies are promising routes for rapid and efficient genetic identification and sequencing of novel pathogens, a prerequisite for public health responses to emerging infectious diseases, and the development of targeted therapeutics and vaccines. The COVID-19 pandemic caused by the severe acute respiratory syndrome coronavirus 2 (SARS-CoV-2, a positive single-stranded RNA virus) has created an unprecedented demand for genetic sequencing and testing. Current techniques for clinical detection of SARS-CoV-2 use nucleic acid (NA) amplification tests (NAAT) including reverse transcription-polymerase chain reaction (RT-PCR) and reverse transcription loop-mediated isothermal amplification (RT-LAMP). These techniques amplify specific viral genes and identify the sequence via fluorescence probes, which is capable of measuring down to $10^3$ RNA copies/mL depending on the Ct (cycle threshold) values obtained. Prior to the development of NAAT-based clinical testing kits and protocols for a novel pathogen, it is necessary to know the pathogen-specific sequence fragments to accurately design primers for the target regions. *De novo* sequencing, which sequences a novel genome where a reference sequence is not available for alignment, has largely facilitated the primer design for newly emerging pathogens. Nanopore translocation technology identifies biomolecules via membrane translocation and is suitable for *de novo* sequencing due to its ability to sequence long reads (> 10 kb) [1]. In addition, nanopore-based sequencing has recently emerged as a promising non-fluorescence-based platform for the testing of SARS-CoV-2. For example, Oxford Nanopore Technologies (ONT), which is a commercial front-runner in translocation-based sequencers, introduced a kit for their MinION system specifically for detecting SARS-CoV-2 by combining nanopore translocation technology with LAMP to validate results from LAMP, since it is prone to false-positives [2].

Nanopores conducive for NA sequencing can be fabricated within biological membranes, synthetic solid-state thin films, and two-dimensional (2D) materials. In particular, 2D nanomaterials are deemed promising for next-generation nanopore sequencing due to their superior chemical and mechanical stability. The atomic-thinness of 2D materials is length-scale matched to the molecular size of NA, which makes them conducive for enhanced spatial resolution and temporal sensitivity in identifying individual base pairs. The fabrication of nanopores requires highly precise material processing techniques to achieve pore sizes just slightly larger than that of the nucleotides (~ 1 nm); these techniques include focused ion beam (FIB), focused electron beam (FEB), and plasma and electrochemical etching [3].

Nanopore sequencing relies on measuring characteristic electric and/or optical (spectroscopic) signals that are unique to each nucleotide [4]. To date, empirically demonstrated nanopore sequencing can be generalized into three general categories based on their fundamental operating mechanisms: (i) trans-pore/membrane ionic current modulation (TCIM), (ii) transverse (in-plane) current sequencing (TCS), and (iii) spectral analysis. Ionic current measurement detects the modulation in osmotic trans-membrane ionic current as NA translocate through a nanopore from the *cis* to the *trans* chamber. Conversely, field-effect current analysis measures the modulation in electrical current across (in-plane) a membrane as a function of translocation events under an applied voltage bias across the membrane. Spectral analysis measures the local optical emission or scattering nearby a nanopore to identify nucleotides as they electrophoretically translocate through the membrane.

Nanopore translocation is touted to enable future platforms for the discovery, sequencing, and testing of novel pathogens but will require significant materials advancements to address current challenges associated with accuracy, throughput, noise, and excessive translocation speeds. Solid-state 2D nanopores promise enhanced stability, tunability, and sensing fidelity compared to state-of-the-art biological nanopores. In this review, we summarize current materials advances for future nanopore NA detection and sequencing platforms. Materials candidates and how nanopores have thus far been fabricated are also discussed with an emphasis on differentiating their material properties. We discuss emerging alternative and hybrid sequencing modalities enabled by new materials advancements and provide future outlook on nanopore translocation-based nucleic acid sequencing technology.

### ADVANTAGES OF NANOPORE SEQUENCING

#### Viral Pathogen Detection in Non-Laboratory Settings

Currently, RT-PCR is the most common mainstay technique for pathogen testing and is considered the "gold standard" for clinical diagnostics of RNA viruses due to its high reliability [4], [5]. For the detection of RNA viruses such as SARS-CoV-2, the RNA first needs to be reverse transcribed into complementary DNA (cDNA), and then amplified and detected via fluorescent probes (Figure 1a). Alternatively, LAMP, although less versatile than PCR, does not require thermal cycling and is relatively inexpensive and more portable. Within clinical settings, the combination of LAMP/PCR and nanopore sequencing can speed up and improve the accuracy of testing for novel pathogens as well as enable the simultaneous detection of multiple pathogens using a single assay. For example, commercially available nanopore sequencing devices, such as the MinION, GridION, and Flongle have been used to create faster sequencing tests to SARS-CoV-2 [6]–[8]. ONT's LamPORE, whici combines a LAMP-based assay with either MinION or GridION, can detect the presence of SARS-CoV-2 from clinical samples in under two hours and can test up to 786 samples on a single flow cell enabling the testing of up to 15,000 samples per day [9]. The precision is enhanced through sequencing three separate regions of the SARS-CoV-2 genome and a control mechanism to distinguish true negatives from false negatives.

Nanopore sequencing is well-established through biological nanopores, as exemplified by ONT devices (Figure 1b). Biological nanopores are formed and harvested from pore-forming bacteria, such as α-hemolysin (aHL), which are then used to measure ionic current modulation to obtain the NA sequence. However, biological nanopores and membranes have limitations due to their sensitivity to pH and temperature. Therefore, solid-state nanopores have become more sought after since they can exceed biological nanopores in possible material candidates, versatile pore morphology, and in enabling new (non-ionic) sequencing modalities (Figure 1c). In particular, 2D membranes exhibit superior material properties conducive for nanopore sequencing such as mechanical robustness, tunable surface morphology, and chemical inertness when compared to their biological counterparts. In addition, the pore sizes of solid-state 2D membranes can be tuned via different fabrication techniques to optimize for high-resolution NA sequencing. Consequently, general improvements have been demonstrated via 2D materials in sensing and noise levels with smaller pore sizes that are similar in size to DNA and RNA fragments (~2 nm).

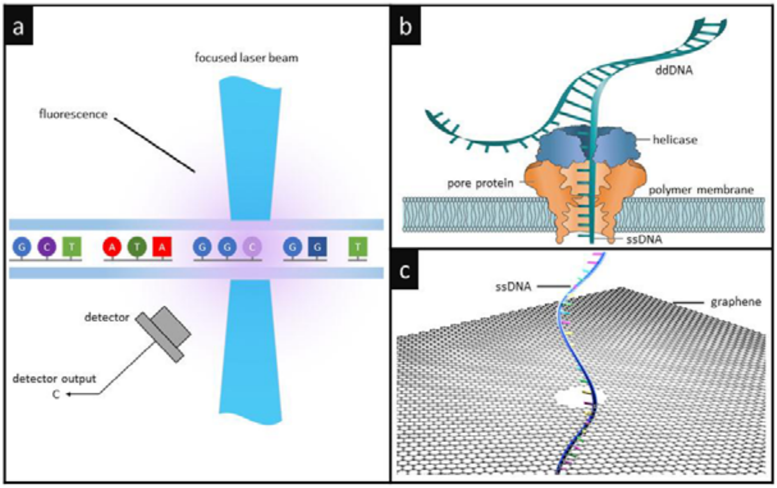

Figure 1. a) Conventional PCR-based techniques detect the presence of pathogens by amplifying the genetic material and uses fluorescent markers to identify the target sequences. b) Nanopore sequencing via protein nanopores identify NA via translocation through the nanopore. Adapted with permission from ref. [10] under Creative Commons Attribution 4.0 International License. c) Solid-state nanopore sequencing enables direct genetic sequencing via translocation through precisely fabricated nanopores in sub-nanometer thick 2D materials such as graphene.

### Whole-Genome Sequencing of Viral Pathogens

The sequencing of whole-genomes is nowadays essential for biological and clinical research of pathogens, for example in the primer design for the development of NAAT-based clinical testing kits [11]. Current state-of-the-art whole genome sequencing techniques are coined as high-throughput sequencing (HTS) or next-generation sequencing (NGS). HTS platforms such as Illumina offer high throughputs for short-read sequencing (up to 600 bp) via the sequencing by synthesis (SBS) technique, which relies on fluorescence-based sequencing [12]. Conversely, nanopore sequencing facilitates long-read sequencing (> 10 kb) without the need for fluorescence, but rather performs sequencing via directly analyzing the translocation-derived (electrical) signals. Importantly, nanopore-based sequencing improves the reference-free assembly, mapping certainty, identification of transcript isoform, and detection of structural variants (i.e., sequence changes > 50bp) compared to short-read sequencing. In addition, nanopore translocation sequencing offers easier operability as well as portability conducive for widespread use in non-traditional and resource-constrained settings. For instance, ONT's MinION device is operable in atypical laboratory settings, starting with accuracy rates of 60% and improving to more than 99% with the use of various bioinformatics pipelines [1], [13], [14].

For sequencing the whole genome of RNA viruses such as SARS-CoV-2 using a conventional pipeline, the RNA first needs to be reverse transcribed into complementary DNA (cDNA). Conversely, nanopore sequencing has the potential to directly sequence RNA strands, rather than having to first transform RNA into cDNA for amplification. Direct RNA sequencing using nanopore arrays allows longer sequencing reads without amplifying the genetic material which can cause copying errors. This has been demonstrated with nanopore sequencing of the longest RNA-based virus (HCoV-229E, ~30,000 nt), which showed the ability to sequence 99.86% of its genome [15].

## MATERIALS AND FABRICATION TECHNIQUES

### Solid-State Membrane Materials

A nanopore - a nanometer-scale perforation in a membrane - can either be intrinsically present in biological proteins/membranes or artificially fabricated in synthetic solid-state materials. Much advancement has been made on a myriad of solid-state materials for fabricating well-defined nanopores with precise morphology, quantity, and pore size distribution (PSD). Compared to biological nanopores, solid-state materials (e.g., dielectric [silicon compound] thin films, 2D materials, etc.) exhibit robust mechanical and chemical stability, high thermal tolerance, and are amenable for scalable manufacturability [16]. However, solid-state material still faces several challenges for NA sequencing. For instance, silicon nitride ($SiN_x$), a widely studied material for nanopore sequencing, has shown limited spatial-temporal sequencing resolution due to its relatively large thickness.

Compared with conventional solid-state thin-film materials, atomically-thin 2D materials have emerged as the state-of-the-art material candidates for nanopore sequencing membranes due to their ångström-scale thinness, high chemical stability, mechanical strength, impermeability, and varied electrical properties (spanning electrically insulating to superconducting). The ultimate-thinness of 2D materials (~0.34 nm for monolayer graphene) is comparable to the distance between adjacent nucleotides (~0.34 nm) in a NA strand [17]. As such, the ångström-thinness of 2D materials enhances the spatial resolution for nanopore sequencing (i.e., precise identification of different base pairs/nucleotides) and can significantly improve the signal-to-noise ratio (SNR) compared to other solid-state and biological nanopores. For ionic current measurements, this can be quantified by the ionic conductance (G) for a solid-state nanopore:

$$G = \sigma\left[\frac{4L}{\pi d^2} + \frac{1}{d}\right]^{-1}$$

Here, σ, d, and L are the electrolyte conductivity (S/m), the nanopore diameter (m), and the membrane thickness (m), respectively [18]. The first and second terms in the bracket denote the channel and access resistance, respectively. Therefore, the high detection sensitivity from the ultimate atomic-scale thinness (L) and precise definition of pore shape and diameter (d) endows 2D materials for nanopore sequencing. Moreover, intrinsic impermeability and excellent mechanical and chemical properties reinforce the advantages of 2D materials for translocation nano-based biosensing [19].

To date, various 2D materials have been used experimentally to realize nanopore NA sequencing including 2D semi-metallic graphene and its derivatives (e.g., graphene nanoribbons), insulating hexagonal boron nitride (hBN), semiconducting transition metal dichalcogenides (TMDC) (e.g., molybdenum disulfide [$MoS_2$], tungsten disulfide [$WS_2$]), and transition metal carbides or nitrides (MXenes) (Figure 2a-d) [20]–[22]. Approaches for the synthesis of 2D materials has progressed rapidly over the past few years to include exfoliation (mechanical, liquid, and gas) and chemical/physical vapor deposition, with ever-improving control over their stoichiometry and morphology [23]. Evolving capabilities to synthesize large-area and low-defect 2D crystals are also enabling future wafer-scale device integrability.

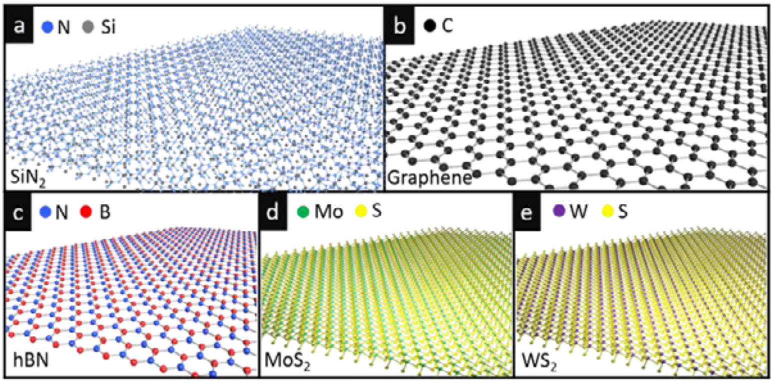

Figure 2. Solid-state membrane materials that have been empirically realized for nanopore sequencing include well-established solid-state materials such as a) silicon nitride ($SiN_x$) and atomically-thin 2D materials such as b) graphene, c) hBN, d) $MoS_2$ and e) $WS_2$.

The prototypical 2D material, graphene, has been widely adopted for *de novo* sequencing, but has earlier suffered from low SNR and low mechanical stability [20]. The π-π interaction of nucleotide strands with graphene is rather strong compared to that with other 2D materials, which causes nuisance adsorption of NA on the graphene basal surface resulting in nanopore clogging and inhibition of translocation [24]–[26]. To overcome these challenges, graphene requires surface-specific functionalization or high ionic strength electrolytes and pH to reduce surface interactions [21], [27]. Moreover, the flexible nature of graphene interferes with precise current measurements during nanopore sequencing due to thermal-mechanical fluctuations.

Therefore, research is pivoting to other 2D materials that exhibit more preferable intrinsic material properties. For instance, $MoS_2$ is a biocompatible semiconductor that displays low off-state leakage current (in FET biosensors) and higher sensitivity compared to semi-metallic graphene [21]. Compared to graphene, hBN and

$MoS_2$ have intrinsic weaker hydrophobicity, which reduces undesirable NA adsorption onto the membrane surface. Similarly, $Ti_2CT_x$ (a 2D MXene) has shown promising nanopore NA sequencing capabilities while exhibiting low SNR, low leakage current, and high sensitivity comparable to $MoS_2$, which is attributable to its hydrophobicity and surface functionalization [22].

### Fabrication Techniques

Conventionally, nanopore sequencing devices warrant device portability with high SNR for long read sequencing [28]. Nanopores intended for NA sequencing should have well-defined and uniform pore geometry, where the nanopore diameter should be small enough to ensure uncoiled/unfolded NA translocation and close to the ångström-width of individual nucleotides (for RNA) or base pairs (for DNA). In addition, the membrane and pore thickness should be comparable to (or less than) the distance between nucleotides to distinguish the sequence. The atomic-thinness of 2D materials allows them to obtain high spatiotemporal resolution for NA sequencing, an intrinsic obstacle for (thicker) conventional thin-film solid-state materials.

Another material prerequisite for rapid and massively-parallel nanopore sequencing is the synthesis of large-scale, uniform nanopore arrays with individually addressable signal measurement. State-of-the-art techniques for fabricating nanopores in solid-state 2D membranes include vacancy nucleation/drilling via focused energy beams (with electrons [FEB] or ions [FIB]), electric pulse ablation, plasma etching, and (electro)chemical etching. Focused energy beam techniques offer precise spatial resolution down to the sub-nanometer scale, with control over the geometry of the nanopores. Focused energy beams perforate the membrane material by generating localized high pressure, temperature, and momentum transfer to induce atom-by-atom sputtering. Typically, FEB is performed in a transmission electron microscope (TEM) with high operating voltage to achieve the required knock-on damage (KOD) threshold which is specific to the particular 2D material (e.g., ~86 keV for graphene) (Figure 3a) [29]. FIB operates at lower acceleration voltage (10-35 keV) compared to FEB as ions are heavier than electrons (Figure 3b). The resolution of the as-fabricated nanopores in the 2D materials is dependent on the probe diameter, current/dosage, operational condition, and exposure time. For FIB, the types of ions (e.g., Ar, He, Ga) along with the applied voltage dictate the ion probe resolution/diameter [30]. To achieve sub-nanometer pore fabrication and to avoid damage on the 2D material surface, light and smaller He ions are favored compared to heavier and larger Ga ions. Additionally, the presence of ambient contamination also plays a role during FEB/FIB and affects the precision, quantity, and edge morphology/chemistry of the pores within 2D membranes. Moreover, combining FIB and FEB has been shown to yield highly precise 2D nanopores [31]. In this hybrid modality, FIB is used to first perforate the membranes to nucleate the nanopore, which is subsequently shrunk to the desired diameter using subthreshold FEB irradiation to achieve localized annealing. Alternatively, in another hybrid modality, FEB perforation of nanopores is followed by dilation via laser irradiation (i.e., photo-oxidation) in the electrolyte to control the final nanopore diameter [32].

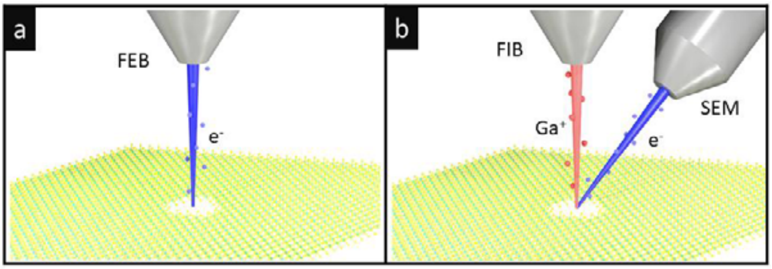

Figure 3. Empirically used nanopore fabrication techniques in 2D materials for nucleic acid sequencing: a) focused electron beam (FEB) fabrication uses an electron beam and b) focused ion beam (FIB) uses ions (typically $Ga^+$ or $He^+$) to perforate 2D materials.

Other techniques such as the electric pulse ablation (electroporation) or controlled dielectric breakdown (CDB) have been investigated in the rapid fabrication of sub-nm resolution nanopores applicable to nanopore sequencing. Here, precise quantities and dimensions of nanopores can be patterned via applying a set breakdown voltage across the membrane and a small voltage to detect the successful fabrication of nanopore [33], [34]. Although this method is inexpensive and does not require vacuum instrumentation compared to FEB/FIB, it lacks control over the exact number of nanopores generated and lacks precise spatial control of the nanopores. Another strategy is plasma/chemical etching, in which controlled exposure of the membrane to energetic plasma (e.g., O, Ar, $H_2$, etc.) or selective chemical etchants can achieve nanopores in 2D materials. However, plasma/chemical etching comes with caveats such as the requirement for pattern definition prior to masked exposure of the 2D material to the plasma or chemical etchants for subsequent (selective) materials removal.

Altogether, focused energy beams (FEB and FIB) are thus far the most explored and viable solutions for nanopore drilling due to their generalizability to arbitrary 2D materials with excellent control over sub-nanometer pore geometry and spatial definition, which are amenable to bespoke nanopore sequencing devices. However, further advances in 2D materials processing are required to synthesize large arrays of monodisperse, highly uniform nanopores towards massively-parallel translocation sequencing platforms.

### SEQUENCING MODALITIES

A key differentiator between various nanopore sequencing techniques stems from their fundamental detection modalities of the NA nanopore translocation events. The prototypical configuration for NA nanopore sequencing consists of an impermeable membrane that compartmentalizes an electrolytic solution with a driving force (e.g., electrical voltage) across the membrane. This electrophoretically drives NA to translocate through the nanopore from the negatively biased *cis* to the positively biased *trans* compartments. During the translocation events, the complex interactions between the translocating NA and the nanopore can be measured in various ways. In this section, we discuss three of the most common modalities that have thus far been realized empirically and are deemed most promising for future development.

### Trans-Pore Ionic Current Modulation (TICM)

Trans-pore ionic current modulation was first employed via biological nanopores and has been commercialized by ONT [35]. The fundamental mechanism for nanopore sequencing via TICM is through analyzing the ionic conductance modulation and translocation dwell time unique to different nucleotides as they electrophoretically translocated through a nanopore from the *cis* to *trans* compartments (Figure 4a) [36].

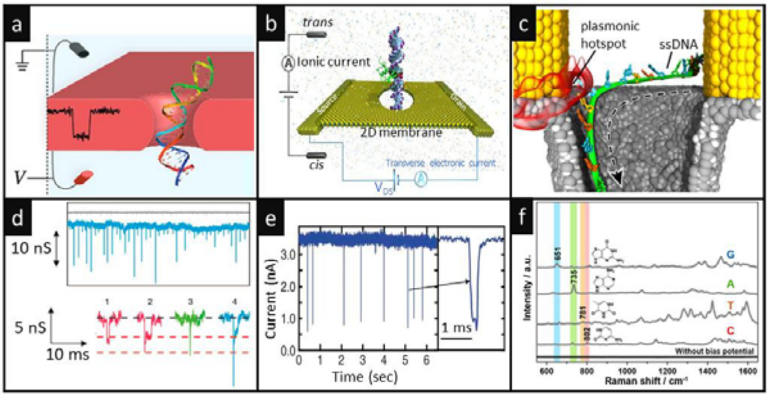

Figure 4. Schematics and measurements of the various detection modalities for nanopore sequencing: a) Trans-pore ionic current modulation (TICM) works on the principle of measuring changes in the trans-pore ionic conductance (through the membrane) during nucleic acid translocation events. Adapted with permission from ref. [37], Copyright [2013] American Chemical Society. b) Transverse current sequencing (TCS) measures the in-plane (across the membrane) transverse current modulation during NA translocation events. Adapted with permission from ref [38]. c) Optical spectral analysis measures the near-field optical signals unique to the base pairs via fluorescence, Raman, etc. d) During TCIM, sequences of translocating nucleotides are identified from the time-varying ionic current modulation. Adapted with permission from ref. [24], [39]. Copyright [2014, 2015] American Chemical Society. e) During TCS, nucleotides sequences are identified from the time-varying transverse current modulation. Adapted with permission from ref [4], Copyright [2010] John Wiley and Sons. f) For spectral analysis, nucleotides sequences may be identified by measuring time-varying changes in the intensity and shift of the characteristic Raman signatures. Reprinted with permission from [40]. Copyright [2019] American Chemical Society.

The sensitivity of the ionic current modulation improves with thinner membranes. Therefore, in comparison to thin-film solid-state membranes made from silicon and silicon compounds ($SiN_x$), atomically-thin 2D materials are ideal for sequencing via ionic current modulation. Common challenges for high-resolution translocation reads include the excessive and difficult to control translocation speeds of NA through the nanopores as well as the nuisance interactions between NA and the 2D materials. Variables such as electrolyte viscosity and concentration gradients have been empirically investigated to improve the probability of NA capture and to slow their transportation rate [41]. In addition, electrolytes with specific cationic species and higher concentrations can reduce the NA translocation speed. Here, different cations impose differential interaction strengths (i.e., higher for Li+ compared to $Na^+$ and $K^+$) for the NA to achieve overall effective charge reduction and increase the stall force on the NA [42]. Other factors to control NA translocation speed include modifying the NA molecule itself using foreign macromolecules, magnetic or optical tweezing, and nanopore size reduction to increase translocation resistance [3], [43]–[46].

For graphene-based TCIM, effective functionalization or specific electrolyte preparation is required to overcome the strong interaction between graphene and NA [47]. In more recent studies, nanopores in $MoS_2$ and hBN membranes have demonstrated higher effectiveness for nanopore sequencing as they exhibit less interaction with NA and thus, are more agnostic to particular sample and electrolyte preparations [19]. Although an electrically insulating membrane has been deemed to reduce the high-frequency noise levels during TCIM, hBN is not preferred due to its lower detection resolution [19], [48]. In addition, $MoS_2$ exhibited low off-state leakage current and a lower device failure rate (<30%) during experimentation (membrane leakage or pore-clogging) compared to graphene [21].

Besides high-resolution detection of nucleotide sequences, TCIM can also distinguish different conformations of the NA translocation event via analysis of the differential ionic conductance [24], [49]. For example, variations in ionic conductance through a $MoS_2$ nanopore can be observed corresponding to different DNA translocation (entry/exit) conformations including 1) completely unfolded, 2) folded entry and unfolded exit, 3) completely folded, and 4) bumping of DNA on nanopore (Figure 4d). Further understanding of the conformation dynamics of NA translocation through nanopores is needed to provide a better understanding of the exact NA translocation process to guide experimental and materials design.

### Transverse Current Sequencing (TCS)

Conventional TCIM is often hindered by excessive NA translocation speed of (0.01-1 μs/base) and a high sampling rate (often over MHz) [50]. In addition, TCIM has high access resistance that makes the sensing length of nanopore larger compared to the actual membrane thickness. To overcome these challenges, an alternative or complementary modality is to measure the in-plane transverse current (TCS) during NA translocation, which is modulated by the changes in the local density of states near the nanopore, in order to identify individual nucleotides [50]. Typically, transverse currents measured (via deposited metallic electrodes) are on the order micro-to-milliamperes range (compared to the picoampere ranges for ionic current modulation), thereby enabling high SNR and high bandwidth measurement at fast translocation speeds. Overall, TCS is analogous to field-effect modulation in transistors (FET), in which the NA modulates the channel (membrane) gating by switching the nanopore states during the translocation events (Figure 4b) [51]. Measurements of the transverse current and the ionic current are independent of one another and can be obtained simultaneously to corroborate the identification of the translocation sequence (Figure 4e) [50].

Insulating solid-state nanopores such as $SiN_x$ membranes are typically precluded from TCIM since transverse currents require semiconducting membrane materials. Due to their exceptional tuneable electrical charge transport and structural properties, an emergent library of 2D semiconductors in addition to semi-metallic graphene are favorable for TCS. For instance, 2D materials have exhibit lower SNR (~30%) for TCS compared to TCIM [50], [52]. Although 2D device fabrication and operation (in the form of nanoribbons, nanogaps, nanotubes) is currently challenging compared to the much better established silicon-based thin films, the rapid advancement of 2D materials fabrication techniques is progressively facilitating novel implementations of TCS.

### Spectral Analysis

Nanopore sequencing via spectral analysis techniques includes surface-enhanced Raman scattering/spectroscopy (SERS) and tip-enhanced Raman scattering (TERS). Conventionally, SERS is primarily used for detecting very low concentrations of distributed biomolecules, while the related TERS technique enables spatially resolving very small or immobilized features [53]. Both techniques involve the use of plasmonic (metallic) nano-features to generate localized excitation and enhance the inelastic Raman scattering from the individual nucleotides near the plasmonic hotspots.

Spectral detection via SERS utilizes plasmonic nanoparticles on or adjacent to the nanopore to induce optical forces on translocating NA and can identify nucleotide sequences with high sensitivity, spatial resolution, and enable precise control on the mobility of biomolecules during nanopore sequencing (Figure 4c and 4f) [39], [54]. For example, the combination of a plasmonic substrate and nanoparticles has demonstrated that NA can be adsorbed on Au nanoparticles for single base detection [55]. Typically, SERS is a non-destructive technique and can be combined with the aforementioned TCIM and TCS techniques to perform simultaneous multimodal nanopore sequencing [56]. On the other hand, detection via TERS is not necessarily dependent on translocation-based detection; rather, it relies on stable NA alignment/deposition on a plasmonic substrate and identification of individual nucleotides via a rastered plasmonic nanotip (single point SERS) [57]. Altogether, coupling state-of-the-art 2D materials with plasmonic nanostructures can enable hybrid nanopore sequencing modalities with high-resolution sensitivity, specificity, dwell time, detection rate, and large-scale parallel detection schema [58], [59].

Additionally, incorporating plasmonic nanostructures with biological nanopores can introduce hybrid modalities [60]. Computationally, plasmonic nanoparticles have exhibited the ability to act as a nanoscale heater and thermometer. In conjunction with a biological membrane, plasmonic nanoparticles can induce localized heating to control the temperature near the nanopore, this increasing ionic conductivity. This coupled modality warrants further experimental validation and device implementation.

## FUTURE OUTLOOK FOR NANOPORE SEQUENCING TECHNOLOGIES

State-of-the-art nanopore sequencing offers advanced translocation schemes, but challenges and limitations are still prevalent. Currently, nanopore sequencing often generates errors and introduces unwanted noise [60]. Additionally, nanopore sequencing is often limited to DNA and RNA, where sequencing of other biomolecules, such as proteins, is considered more challenging since it requires distinguishing 20 different amino acids compared to the four bases in DNA sequencing [61]. Besides, entire protein sequencing typically requires denaturation; therefore, solid-state nanopores are potentially suitable and preferable over biological nanopores due to their lower sensitivity to environmental/experimental conditions.

Typically, a higher conductance output (and large modulation between different nucleotides) with a high SNR is required to achieve high-resolution nanopore sequencing. The low-frequency 1/f noise (‘flickering’ noise) level dictates the SNR and the detection limit of nanopore sequencing devices, which can arise from the transient electrolyte ion trapping-detrapping process on the inner surface of nanopores, mechanical and thermal fluctuations, impurities in nanopores, and surface charge fluctuations [22], [27], [61]. Moreover, the output conductance and the SNR depend on various experimental conditions including the dynamic conformational and charge states of the target NA, the electrolyte concentration, solution temperature, bias voltage, pH, bubble formation in the nanopore channel, nanopore size, morphology and chemical functionalization or presence of dangling bonds of nanopore surfaces [62]–[64]. As discussed, these sources of noise in the output conductance require appropriate modulation and control to achieve the highest SNR, ultimate spatial-temporal resolution, and error-free nanopore sequencing, which warrants further research.

To date, many strategies to improve nanopore sequencing have been explored, including introducing novel hybrid modalities for NA identification through functionalization, doping, and heterostructures of nanopores and 2D materials. Hybrid solid-state/biological nanopores containing a biological nanopore placed inside a solid-state nanopore have been explored computationally and showed long device lifespan and stability under various environmental conditions that warrant empirical validation [67]. In addition, solid-state nanopores can be combined with optical techniques to hybridize ionic-current with force modulation modality. Here, to probe the forces involved during the translocation process, the translocation speed and force can be controlled by an optical tweezer or anchoring to an AFM tip [68], [69]. The turnstile-like motion of NA provides well-defined translocation kinetics to be correlated with the individual nucleotides, however, has yet to be determined experimentally with 2D membranes.

Another innovative way of modulating the interaction between the NA and solid-state nanopores is by introducing NA origami to selectively functionalize and alter the chemical properties of the nanopore [47]. Computational studies have shown that by decorating dangling bases or placing sheets of NA, the induced higher interactions (due to hydrogen bonding) can control and reduce the speed of the NA translocation through the nanopore (Figure 5a) [70].

The plethora of 2D materials introduces many membrane material choices with tunable mechanical, electrical, and (electro)chemical properties. For example, doping can introduce site sensitivity in the 2D material lattice, which in turn may increase sequencing sensitivity by modulating effective conductance. Besides, deterministic nanoscale surface deformations (e.g., crumpling or wrinkling) can modulate the Debye lengths, which lowers the shielding of biomolecules and increases the detection sensitivity (Figure 5b) [71]. In addition, the atomically-thin nature of 2D materials make them conducive for novel device architectures. For example, the π-π interaction between a sliding NA molecule (parallel to the basal plane) and graphene (in a nanofluidic, nanochannel device) can distinguish the Fano resonance-based conductance for individual nucleotides to perform sequencing (Figure 5c) [73]. However, such nanochannel-based DNA sequencing architecture has yet to be experimentally realized.

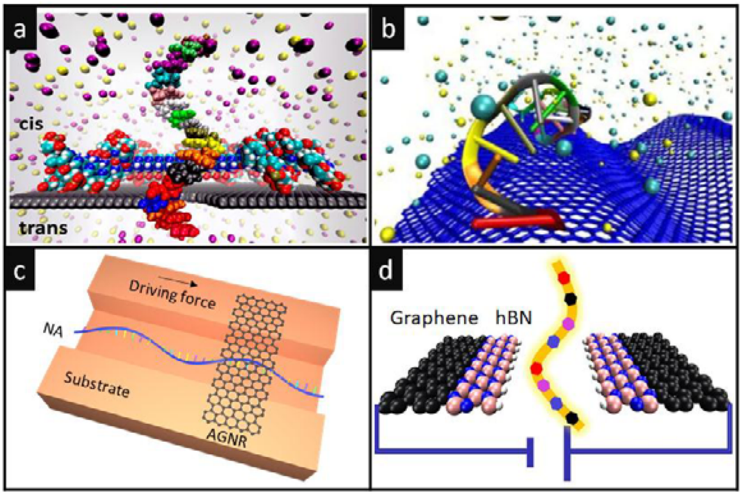

Figure 5. Outlook on materials advances in nanopore sequencing: a) Schematic of a hybrid nanopore which consists of DNA origami on graphene. The site-specific functionalization modulates the graphene–nucleic acid interaction and translocation speed. Adapted with permission from ref. [47]. Copyright [2017] American Chemical Society. b) Deterministic nanoscale deformation (i.e., crumpling/wrinkling) of graphene modifies the Debye screening length which can increase the detection sensitivity. Adapted with permission from ref. [71] under Creative Commons Attribution 4.0 International License. c) Schematic of NA moving through a nanochannel where individual nucleotide detection is realized through Fano resonance-based conductance modulation. d) Graphene-hBN heterostructure reduces the edge reactivity of graphene to modulate graphene-nucleic acid interaction. The vertical stacking and in-plane lateral heterojunctions mitigate nanopore clogging and reduce the device failure rate. Reprinted with permission from ref [72]. Copyright [2017] American Chemical Society.

Heterostructures of different 2D materials can also lead to higher sensitivity and performance. Graphene-hBN heterostructures with vertical stacking and in-plane lateral heterojunctions subdue the edge reactivity of graphene to reduce interaction which mitigates the tendency of nanopore clogging (Figure 5d) [72]. Analogously, graphene-$MoS_2$ heterostructures have exhibited the capability for protein sequencing, making this architecture viable for NA sequencing [74]. Overall, these 2D material heterostructures have been explored primarily via computational modelling, and there exist many 2D materials combinations and heterostructure permutations which exhibit immense potential for future NA sequencing architectures.

### CONCLUSION

Translocation-based nanopore sequencing technologies offer facile, fast, and effective nucleic acid (NA) sequencing with the potential for *de novo* sequencing for epidemiological and biological research of currently evolving (e.g., SARS-CoV-2) and future emergent pathogens. In this review, we have summarized the current status, recent advancements, and future outlook of the state-of-the-art 2D nanomaterials based nanopore NA sequencing techniques. Compared to biological nanopores, solid-state nanopore sequencing has enabled high sensitivity NA detection with robust mechanical and chemical stability. With ångström-scale thickness, excellent and tunable structural, chemical and electrical transport properties, 2D materials are promising solid-state membranes for translocation-based NA sequencing. Various existing as well as emerging techniques are conducive for the fabrication of 2D materials with well-defined nanopores with controllable size and geometry, which make 2D materials suitable for technological adoption and further research for NA sequencing. Several optoelectrical 2D nanopore sequencing modalities have already been experimentally demonstrated, with further research needed to overcome challenges such as detection accuracy and noise level towards truly facile, rapid, portable, and reliable nanopore-based NA sequencing schemes.

### ACKNOWLEDGEMENT

BAS, MRT, YJJ, and MCW acknowledge support from the University of South Florida New Researcher Grant (NRG), the University of South Florida Pandemic Response Research Network (PRRN) COVID-19 Rapid Response Grant, and the ORAU Ralph E. Powe Junior Faculty Enhancement Award. This material is based upon work supported in part by the National Science Foundation under Grant No. 1944638.
